# Free HIV Antiretroviral Therapy Enhances Adherence among Individuals on Stable Treatment: Implications for Potential Shortfalls in Free Antiretroviral Therapy

**DOI:** 10.1371/journal.pone.0070375

**Published:** 2013-09-05

**Authors:** Jayne Byakika-Tusiime, Eric C. Polley, Jessica H. Oyugi, David R. Bangsberg

**Affiliations:** 1 Department of Epidemiology and Biostatistics, School of Public Health, Makerere University College of Health Sciences, Kampala, Uganda; 2 Biometric Research Branch, National Cancer Institute, Bethesda, Maryland, United States of America; 3 Infectious Diseases Institute, Makerere University College of Health Sciences, Kampala, Uganda; 4 Department of Medicine, Massachusetts General Hospital for Global Health, and Harvard Medical School, Boston, Massachusetts, United States of America; McGill University Health Centre, McGill University, Canada

## Abstract

**Objective:**

To estimate the population-level causal effect of source of payment for HIV medication on treatment adherence using Marginal Structural Models.

**Methods:**

Data were obtained from an observational cohort of 76 HIV-infected individuals with at least 24 weeks of antiretroviral therapy treatment from 2002 to 2007 in Kampala, Uganda. Adherence was the primary outcome and it was measured using the 30-day visual analogue scale. Marginal structural models (MSM) were used to estimate the effect of source of payment for HIV medication on adherence, adjusting for confounding by income, duration on antiretroviral therapy (ART), timing of visit, prior adherence, prior CD4^+^ T cell count and prior plasma HIV RNA. Traditional association models were also examined and the results compared.

**Results:**

Free HIV treatment was associated with a 3.8% improvement in adherence in the marginal structural model, while the traditional statistical models showed a 3.1–3.3% improvement in adherence associated with free HIV treatment.

**Conclusion:**

Removing a financial barrier to treatment with ART by providing free HIV treatment appears to significantly improve adherence to antiretroviral therapy. With sufficient information on confounders, MSMs can be used to make robust inferences about causal effects in epidemiologic research.

## Introduction

Access to antiretroviral therapy (ART) continues to expand at a rapid rate [1], [2], [3]. Of the estimated 9.5 million people in need of treatment in 2008 in low- and middle-income countries, 42% had access, up from 33% in 2007 [3]. The greatest progress was seen in sub-Saharan Africa, where two-thirds of all HIV infections occur. Prices of the most commonly used antiretroviral drugs have declined significantly in recent years, contributing to wider availability of treatment. In most cases ART is provided at no cost to patients. [3]. There is substantial concern, however, that there are insufficient resources available to continue the scale-up of free antiretroviral therapy to all that need it (NYT article on Uganda, 2009). Insufficient resources for the steady supply of new patients initiating treatment in most resource-limited settings may require that patients once again pay for ART. How will the potential reintroduction of self-pay therapy impact adherence in settings where there is an inadequate supply of free therapy?

Many studies in resource limited settings have documented that the cost of medications is a major predictor of non-adherence to ART [4], [5], [6], [7], [8] All these studies were observational in design. While important associations between variables can be obtained from observational studies, such studies often are unable to adequately control for confounding, leading to biased estimates of causal effects. In observational studies, estimation of the causal effect of an exposure on an outcome may be biased because of confounding, i.e. covariates associated with treatment may also be associated with the potential response, so that the observed response differences cannot be attributed directly to the exposure. Proper estimation of causal effects must account for confounding. In studies where the treatment/exposure does not change (i.e. point treatment), the traditional method of analysis is to model the probability of disease as a function of exposure and pretreatment covariates. However, with a time-varying exposure, these traditional methods may be biased if time-varying covariates are simultaneously confounders and intermediates-that is, if covariates are predictors of the outcome and also predict subsequent exposure, and past exposure history predicts resulting covariate level [9]. Such covariates are called time-dependent confounders [9], and they pose unique analytical challenges requiring specialized methods.

We used marginal structural models (MSMs) using a targeted maximum likelihood estimate (MLE) to estimate the causal effect of source of payment for ART on treatment adherence among HIV-infected individuals in Kampala, Uganda from 2002 to 2007. This was a time of rapid transition from exclusively self-pay to free HIV ART. Marginal structural models (MSMs), developed by Robins et al [9], [10] can obtain causal effect estimates in observational studies [11], [12], [13], [14], where causal effects are typically defined as the population exposure of interest changes, such as payment source of ART from self pay in 2002 to free in 2007. These models are appealing because the coefficients are directly interpretable causally and they provide unbiased marginal estimates, even in the presence of time-dependent confounding. Hence, the aim of this analysis was to estimate the population-level causal effect of source of payment for ART on treatment adherence using MSM and to compare the MSM estimate with estimates from traditional statistical models. MSMs can be used for causal inference unlike traditional models that suffer confounding effects.

## Materials and Methods

We utilized data from the Adherence Monitoring Uganda (AMU) study [15], [16]. AMU was an observational prospective cohort study of adherence and treatment response among individuals on HIV generic antiretroviral therapy conducted from 2002–2007 in Kampala, Uganda. The cohort, assembled from patients initiating ART from several treatment centers in Kampala, was comprised of patients on self-pay and those on free treatment. During the study period some subjects switched from self-pay to free treatment. Social-demographic characteristics, source of payment for antiretroviral therapy, HIV RNA and CD4 cell count were obtained prior to initiating antiretroviral therapy. Participants were then followed prospectively to determine source of antiretroviral therapy, adherence, HIV RNA, and CD4 cell count every month for 6 months and then every 3 months for up to 18 months. Antiretroviral adherence was estimated using 4 measures: 3-day structured self-report (1- number of doses reported missed/doses prescribed over the prior 3 days), 30-day visual analogue scale (1- percent of pills reported missed over the last 30 days), electronic medication monitoring (number of pill bottle openings registered/number of doses prescribed), and unannounced monthly pill counts (1- number of pills missing between counts/number of pills prescribed between counts). Correspondence between the 4 measures was compared. For this analysis we used the 30-day visual analogue scale measurements because the other 3 measures were discontinued after 6 months of follow up for each participant when interim results showed that all 4 measures were closely correlated with each other (R = 0.77–0.89) [15] At each visit, participants were asked who paid for their medications. Additional details of the recruitment and follow-up of patients have been previously described [15], [16].

### Statistical analyses

Marginal structural models (MSM) were used to estimate the difference in adherence means for a given month that would have been observed between the treatment group (those individuals that received free therapy) and the control group (those individuals who paid for their therapy) if source of payment for therapy had been assigned randomly. The marginal treatment effect is the parameter of interest. A targeted maximum likelihood estimator (TMLE) was used to estimate this parameter. A data set was created that consisted of a data point for each person-month during follow-up for which source of payment for medication and subsequent adherence were measured. Confounders considered included prior adherence, prior CD4 T cell count, prior plasma HIV RNA level, income, duration on ART and time of visit (period from study enrolment when study staff visited participant and assessed adherence).

#### MSM Assumptions

**Figure 1:**
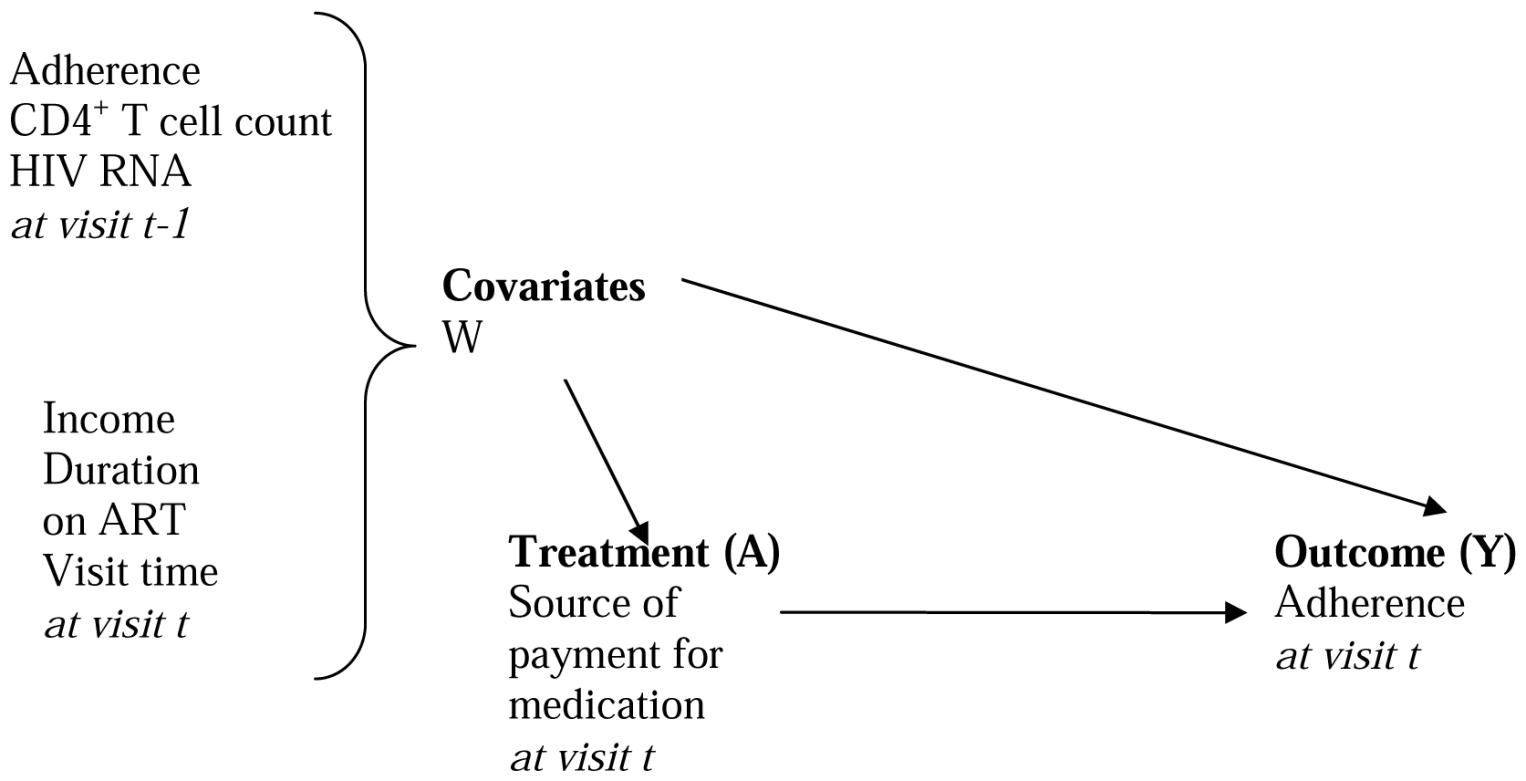
Directed acyclic graph (causal diagram).

Several assumptions were made in order to use the MSM to estimate the parameters of interest. *Counterfactual assumption:* we assumed that counterfactuals exist and that the outcome observed for each patient was one of the potential outcomes. We thus assumed that this was a missing data problem. In conjunction with the counterfactual assumption, we assumed that the treatment or exposure was independent of the counterfactual outcomes given the covariates *(Randomization assumption)*. That is, there were no unknown confounders (NUC). We are not very certain that all confounders were identified. However, using a directed acyclic graph (DAG) we tried to identify the important confounders and these were included in the analysis (Fig. 1). Given that this was a secondary data analysis that was not planned for when designing the study, many important variables/confounders may have been missed. *Time ordering assumption:* The data collection procedures ensured that the time ordering assumption was met. That is, the potential confounders for the relationship between the treatment at a given time, *t*, and the outcome at that time existed prior to the treatment.

*Correct model specification:* The Super Learning procedure was employed to select the best model [17], [18]. Super Learner is a statistical analysis package that comprises different algorithms and selects different algorithms for each application. It reduces variances and improves standard errors. A targeted maximum likelihood estimator was then used to optimize the bias/variance tradeoff for the target. Thus, we assume that our model specification was correct.

*Experimental Treatment Assumption (ETA):* We assumed that treatment was not assigned deterministically based on prior conditions. The probabilities of receiving the treatment were between 0.2 and 0.6 suggesting that the ETA assumption was met.

#### Causal effect estimation

The marginal effect of treatment (causal effect) was estimated by calculating the difference between 1) the mean outcome when all patients was assumed to have been on free treatment and 2) the mean outcome when all patients was assumed to be on self-pay treatment. Targeted maximum likelihood estimation (TMLE) was used to reduce bias in the parameter of interest [19]. First, the mean of the distribution of the outcome [adherence at a given visit] was estimated using the Deletion/Substitution/Addition (DSA) algorithm [17], [18]. The DSA is a data adaptive procedure that employs a cross validation process on the data where by the data is progressively divided into training and validation samples. Using DSA based on multivariable logistic regression of source of payment for treatment on confounders, the probability of receiving the treatment (treatment mechanism) given one's covariates was then estimated. Time-lagged confounder measurements were used to ensure that confounders occurred before (and, therefore, could not be influenced by) payment source (Figure 1). HIV RNA values and income were log transformed. A function of the treatment mechanism (*clever* covariate) was then calculated [20]. The clever covariate for this particular parameter of interest is the inverse probability of receiving treatment when the treatment is observed and negative the inverse probability of not receiving treatment when the treatment is absent. The *clever* covariate was then used to target the parameter of interest. The targeting step was performed by regressing the outcome (adherence at a given visit) on the *clever* covariate using the initial estimate of the mean adherence level for each individual used as an offset. The coefficient on the *clever* covariate represents the degree of confounding in the parameter of interest. The targeting step is repeated until this coefficient is zero. For this analysis, convergence was attained in one step. Standard errors for calculating the 95% confidence intervals were estimated using clustered bootstrap (i.e. randomly sampling patients with replacement). All analyses were conducted using R software Version 2.7.2.

Parallel analyses to estimate the effect of treatment were conducted using generalized estimating equations (GEE) and ordinary least squares (OLS).

## Results

### Participant characteristics

Seventy-six participants were included in the analysis with a total of 251 observations. The 76 participants were observed for a total of 1669 person-months. Median follow up time was 22 person-months (IQR 18–27). Participants initiated therapy at advanced stages of HIV infection, with a mean CD4 cell count of 56 cells/ml [SD 130] and median log_10_ copies RNA/ml of 5.53 (IQR 4.91–5.82). The majority of the cohort was female (63.9%). The mean age was 36 years (SD 7.5). Fifty-five percent of the participants earned less than 60 US dollars a month. One third of the study participants had completed up to a primary level of education. Details of participant characteristics at study entry have been published elsewhere [16]. Half of the participants switched from self-pay to no cost therapy (38/76).

### Predictors of treatment

In the model for the treatment mechanism, receiving free treatment was more likely to occur among individuals with a lower prior CD4^+^ cell count and at later visits (Table 1).

**Table 1:** Multivariable regression model of source of payment for HIV medication on confounders.

Term in multivariable logistic regression model	OR (95%CI)
Prior adherence	1.002 (0.983 to 1.021)
Prior HIV RNA^a^	1.042 (0.878 to 1.235)
Prior CD4^+^ cell count	0.998 (0.997 to 0.999)
Income^a^	0.971 (0.906 to 1.041)
Duration on ART	1.000 (0.998 to 1.003)
Time of visit	1.905 (1.333 to 2.722)

NOTE: Model was selected using cross-validated deletion/substitution/addition algorithm.

a log transformed.

### Adherence and source of payment for HIV medication

**Figure 2:**
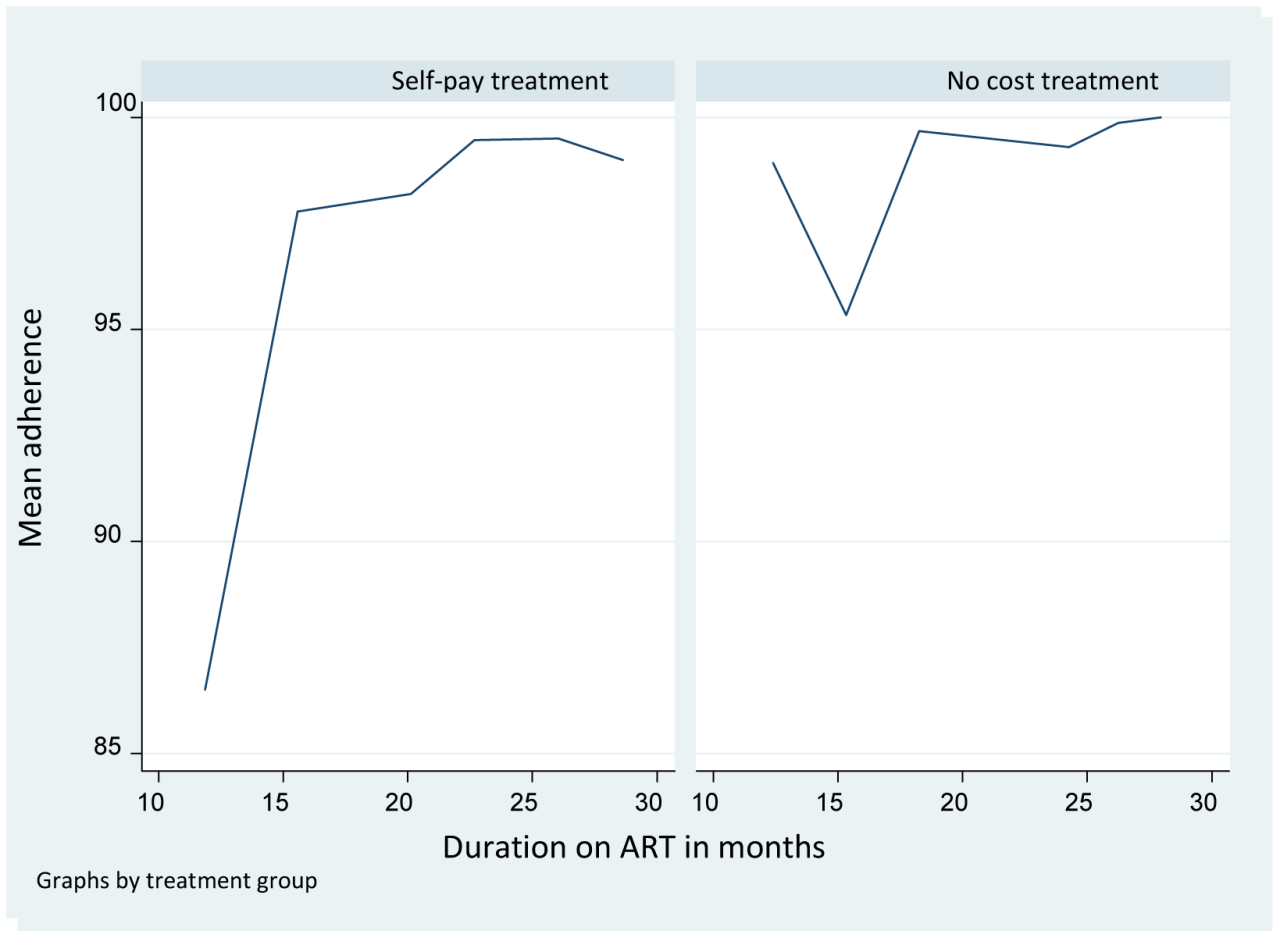
Comparing mean adherence rates for person-months on self-pay treatment and person-months on free treatment.

Overall mean adherence (± SD) over the course of follow up was 95.68%±16% with a median of 100% (IQR 100%–100%). Mean adherence in the self-pay person-months was 93.50%±19.16% while that in the free person-months was 98.56%±9.78% (Figure 2). In the model selected by the DSA algorithm, current higher adherence was more likely to occur among patients with a higher rate of prior adherence and those with lower prior HIV RNA (Table 2).

**Table 2:** Multivariable regression model of adherence percentage on source of payment for HIV medication and confounders.

Term in multivariable linear regression model	Coefficient (95%CI)
Source of payment for HIV medication	3.332 (−0.575 to 7.239)
Prior adherence	0.232 (0.107 to 0.357)
Prior HIV RNA^a^	−1.413 (−2.546 to −0.279)
Prior CD4^+^ cell count	−0.003 (−0.014 to 0.007)
Income^a^	0.054 (−0.406 to 0.514)
Duration on ART	0.003 (−0.014 to 0.020)
Time of visit	1.337 (−0.964 to 3.637)

NOTE: Model was selected using cross-validated deletion/substitution/addition algorithm.

a log transformed.

Applying the targeted maximum likelihood estimator, receiving free HIV medication was estimated to increase adherence by 3.82% compared to self-pay treatment. This was slightly higher than the estimates from two traditional association models - generalized estimating equations (GEE) and ordinary least squares (OLS) (Table 3).

**Table 3:** Marginal structural model estimates vs. Traditional model estimates of the effect of source of payment for HIV medication on adherence.

Method	Difference in mean adherence (95% CI)
Marginal Structural Model	3.82 (0.97–6.66)
Generalized Estimating Equations	
Crude	6.26 (2.66–9.85)
Adjusted*	3.10 (0.95–5.24)
Ordinary Least Squares	
Crude	5.06 (1.09–9.04)
Adjusted*	3.33 (−0.57–7.24)

* adjusted for income, duration on ART, prior adherence, prior CD4+ T cell count, prior HIV RNA.

## Discussion

Using a marginal structural model, we estimated a 3.8% difference in mean adherence when HIV-infected patients receive free HIV treatment compared to when they pay for the treatment out-of-pocket. Our finding confirms other studies finding higher proportion of self-reported adherence [5], [8]. In a cohort in Senegal, adherence rose from 83% to 93% when the cost of HIV medications was reduced [5]. In Cameroon, Boyer et al found an inverse relationship between adherence and self-reported financial difficulties [21]. Weiser and colleagues showed that if cost was removed as a barrier to adherence, the proportion of adherent individuals in Botswana would increase from 54% to 74%] [8]. While all of these studies, including our study, were observational studies, there are some important differences to note. The Botswana and Cameroon studies were cross sectional in design and provide more limitations in their causal inference. Our study employed a marginal structural model for analysis while the other studies employed traditional association models. Causal inference can be made with MSMs unlike traditional models that suffer confounding effects.

The MSM is a semi-parametric model whose validity depends on meeting specific assumptions. However, some of the assumptions cannot be tested from the data. Non-testable assumptions are the time ordering and the counterfactual assumptions. Nevertheless, we are certain that the time ordering assumption was met because three of the authors participated in the design and implementation of the study. We are not very certain that the randomization assumption was met given the limited number of confounders that were collected. Statistical analysis showed that the ETA assumption was met. We believe that our model was correctly specified because we employed *Super Learner* which is the best approach for selecting big models.

All participants in this analysis initiated ART with advanced disease. This was not intentional by the investigators neither was it a choice of the patients. At the time of enrolment of patients into this study, HIV drugs were not readily available and they were very costly. Those few who could access them were supported by family and friends through huge financial sacrifices. As such these patients initiated treatment very late as sustainability of treatment was not guaranteed. However later in the course of the study, the government of Uganda gradually introduced free ART for all HIV patients. Because of the large number of patients, poorer and sicker patients (as per their CD4 count) were often given priority over their fellow patients with slightly better immunity. Another cause for late initiation of ART was stigma. Patients did not want to be identified as HIV positive hence kept away from care until very late when there was no choice but to show up if they wanted to live. It is possible that the results of this study could have been affected by selection bias given that the majority of the study participants were of a low social economic class and hence could not afford medications which consequently led to switch to free ART with priority. However, a qualitative study conducted in the same population to understand how and why the patients had exceptional adherence revealed that the main reason for adhering to ARV medications was the desire to live and take care of other family members [22]. This factor surpassed any other reason. Despite financial constraints, participants rarely reported missing a dose of antiretroviral medication. However, they described this excellent adherence as the product of a constant battle to overcome the barrier of drug cost.

Our analysis using traditional models gave effects of 3.3% and 3.1% using a repeated measures model and the ordinary least squares model respectively. These effect sizes were less than the one from the MSM, underestimating the net effect of payment source on adherence, with the estimate from the ordinary least squares models failing to meet statistical significance (95% CI −0.57 to 7.24) Other analyses have shown greater attenuation of effects and even reversal of effects when using traditional statistical models [23], [24]. The 95% confidence intervals for estimates from all three models [including the MSM] were wide suggesting that the study had a small sample size. The small sample was nevertheless adequately powered to detect the difference with statistical significance [with the MSM and GEE] implying that an association truly exists between source of payment for ART and ART adherence. Larger studies may need to be conducted to establish the precision of this estimate.

There are several of limitations to our study. Our study was conducted among a small sample of ARV-naïve individuals who initiated treatment at an advanced stage of disease. This may limit generalizability of the findings. Although the analysis showed a significant association between source of payment for ART and adherence, there is a possibility that the results could have been biased. The following biases may have occurred although the extent of bias cannot be quantified. Selection bias is very likely to have influenced the results. Study participants were recruited from health facilities in Kampala. However, because of the extent of stigma that many HIV patients suffered at that time, many patients, especially those who could afford to purchase their medications, preferred to meet their care givers in private or to just send another person to the health facility to refill their prescriptions. Consequently, patients that were recruited in the study may not have been representative of all HIV-infected patients who were on ART. As such, the odds of selection for the exposed (those on free-pay) were not equal to the odds of selection for the non-exposed (those on self- pay) hence selection bias. There was an over representation of exposed non-cases (adherent) compared to the non-exposed cases (non-adherent). Information bias was another possible bias. This could have occurred as a result of loss to follow up as 50% of the patients who died (10% of those enrolled) died within 6 months of initiating ART and the majority of these were on self-pay treatment [16]. Another source of information bias may have been due to end-digit-preference in estimating adherence using the visual analogue scale. That is, someone would rather report 80% than 73% or 77%. While the VAS was closely associated with viral suppression and other measures of adherence in this setting, it has performed variably well in other contexts [15], [25], [26], [27]. Confounding bias is also possible in that we did not consider all the possible factors that could likely distort the true relationship between the main predictor and outcome. Possible confounding factors not considered were state of depression and level of education. We did not explore in detail the inherent difference between exposed patients and non-exposed patients irrespective of how they obtained their medications. Patients who paid for their medications may have been different in significant ways from those who received free treatment. We had an insufficient number of HIV RNA determinations to estimate the impact of payment source on viral suppression.

Though useful in establishing causality, the marginal structural model has limitations. It makes the strong assumption of no unmeasured confounders. Causal effects can be estimated from the MSM parameters only if all relevant covariates are measured in the data and are adequately controlled in the analysis, including having appropriate models for determining the treatment mechanism and consequently the *clever* covariate which targets the parameter of interest. The MSM can correctly adjust for *measured* time-varying confounders that are affected by exposure. Given that this was a secondary data analysis that was not planned for when the study was designed, all confounding factors may not have been included in the analysis thus compromising the validity of our findings.

Our study also had several strengths. The study was conducted at a time when the healthcare system in Uganda was transitioning from self-pay to free treatment, which provided a “natural experiment” for study. It would be unethical at the present time to conduct a randomized trial to answer the question addressed in this analysis. Furthermore, state-of-the-art data analyses and the use of alternative methods to control for confounding improved the robustness of the findings.

In summary, we found that receiving free HIV treatment was associated with better adherence among low income HIV-infected patients in a resource-limited setting. In a separate qualitative study conducted in the same population, lack of enough finances to purchase medications was reported as the main reason for missing doses [22]. Our findings are useful in the ongoing discussion on the feasibility of continuing free therapy and related debates as to whether user fees should be introduced in resource-limited settings. There is broad consensus that user fees are an important barrier to accessing health services, especially for poor people [28], [29], [30]. Increasing the cost of care is likely to lower adherence and introduce the possibility of rationing and/or sharing drugs among HIV-infected family members. User fees to secure ART will add to existing structural-economic barriers to care, such as transportation and lost income production, which are significant barriers even with free ART [4], [31], [32]. Increasing the cost of care, through reintroduction of self-pay therapy, may compromise the dramatic success of the ART scale up to date. Further research is needed to understand how adherence changes over time when patients are on free or subsidized treatment. Once the cost factor is removed from the adherence equation, what are the other modifiable factors that influence adherence?
